# Manual federated simulation for multiple sclerosis integrating XGBoost algorithm with SHAP explanation

**DOI:** 10.1038/s41598-026-63991-1

**Published:** 2026-07-30

**Authors:** Hagar E. Ghazy, Zainab H. Ali, Tamer Medhat

**Affiliations:** 1 Department of Artificial Intelligence, Faculty of Artificial Intelligence, Delta University for Science and Technology, Gamasa, Dakahlia 35712 Egypt; 2 Department of Machine Learning and Information Retrieval, Faculty of Artificial Intelligence, Kafrelsheikh University, El-Geish st, Kafrelsheikh, 33516 Egypt; 3 Department of Embedded Network Systems and Technology, Faculty of Artificial Intelligence, Kafrelsheikh University, El-Geish st, Kafrelsheikh, 33516 Egypt; 4 Department of Electronics and Computer Engineering, School of Engineering and Applied Sciences, Nile University, Giza, Egypt

**Keywords:** Multiple sclerosis, Clinically isolated syndrome, Artificial intelligence, Machine learning, Explainability, Federated learning, Computational biology and bioinformatics, Diseases, Neurology, Neuroscience

## Abstract

Multiple sclerosis (MS) is a chronic autoimmune disorder of the central nervous system, underscoring the importance of early and accurate diagnosis. In this study investigates the predictive modelling of MS progression in patients with Clinically Isolated Syndrome (CIS), privacy-preserving for a federated and explainable Machine Learning (ML) framework. To address missing data while preserving inter-feature dependencies, Multivariate Imputation by Chained Equations (MICE) with iterative imputers was employed. Classification was performed using the Extreme Gradient Boosting (XGBoost) algorithm. Model interpretability was developed through Explainable Artificial Intelligence (XAI) techniques, specifically Shapley Additive Explanations (SHAP). To ensure data confidentiality and simulate decentralized clinical environments, an in silico federated learning framework was applied. Experimental results demonstrated strong predictive performance, achieving 96.7% accuracy and 99% ROC–AUC during training, 92.5% accuracy in validation, and 81.8% accuracy with an AUC of 88% on the test set. For the Federated Learning (FL) simulation, the model maintained competitive performance, yielding an accuracy of 76.3% and an AUC of 83.9%. The proposed approach supports early diagnosis, enhances clinical trust through interpretability, and promotes secure data collaboration, thereby contributing to more informed and transparent clinical decision-making and improved patient care.

## **Introduction**

Multiple sclerosis (MS) is a chronic, immune-mediated disorder of the central nervous system characterized by inflammatory demyelination, axonal damage, and progressive neurological dysfunction. Despite advances in therapeutic strategies, MS remains incurable and poses a significant long-term burden on patients’ physical, cognitive, and psychosocial well-being^1,2^. The disease typically follows a relapsing course, with Clinically Isolated Syndrome (CIS) representing the earliest clinical manifestation and often serving as the first neurological event suggestive of MS. CIS is determined by a single episode of neurological symptoms lasting at least 24 h and attributable to inflammatory demyelination. Long-term follow-up studies have demonstrated that a considerable proportion of individuals presenting with CIS subsequently experience recurrent relapses and progress to clinically definite MS, underscoring the critical importance of early risk stratification, accurate diagnosis, and timely intervention^3,4^.

MS is one of the most serious diseases that significantly and negatively impacts human life. It causes the loss of the myelin sheath surrounding nerves, affecting vision, limb movement, and other functions^5^. Therefore, having a model that predicts the progression of a patient’s condition from CIS to MS is crucial for implementing appropriate treatment measures that can limit disease progression and control relapses. Machine learning (ML) is now used in many areas of life, most notably in the medical field^6^. However, maintaining data confidentiality is paramount due to the sensitivity of the data; therefore, integrating ML with Federated Learning (FL) was crucial^7,8^.

Sharing medical data is crucial for scientific research and for comparing results from different cases to enhance research credibility^9^. However, with technological advancements and the increasing reliance on sensors and devices that transmit data, there is a significant risk of cyber threats that could endanger patients’ lives if their data or medical reports are tampered with^10^. When AI models are integrated with medical data, these models require centralized data storage to collect and process patient data on a global server. This exposes them to the risk of easy database breaches and the leakage of sensitive information. Therefore, using FL technology is essential, as it keeps patient data local, trains the model on it locally, and only sends model updates to the global server, ensuring the data never leaves its original storage location^11^.

Despite the development of machine learning models in the medical field, most scientific research still relies on centralized data storage, which threatens data leaks in the event of any technical error or permanent data loss, in addition to the availability of satisfactory results when higher levels can be achieved.

The main objective of this study is to introduce a robust, interpretable, and privacy-preserving ML framework for predicting the progression of CIS to MS. Leverage the predictive power of the XGBoost algorithm while addressing critical challenges in clinical decision-support systems, including data incompleteness, model transparency, and patient data confidentiality. By integrating Multivariate Imputation by Chained Equations (MICE) for reliable handling of missing clinical data, Explainable Artificial Intelligence (XAI) techniques, particularly SHAP for transparent feature attribution, and an in-silico federated learning simulation to enable decentralized and privacy-aware model training, the study seeks to demonstrate that high diagnostic performance can be achieved without compromising interpretability or data security. Ultimately, the objective is to support early MS diagnosis, enhance clinicians’ trust in ML–driven predictions, and facilitate secure collaboration across healthcare institutions, thereby improving patient outcomes and advancing the adoption of trustworthy AI in clinical practice.

The rest of this study is organized as follows: The remainder of this research is organized as follows: Sect.  2 presents background about MS and its integration with AI and FL in relevant work that uses AI techniques in early prediction and diagnosis. Section  3 presents the proposed methodology for machine learning and federated learning models. Section  4 represents the experimental results and the dataset used. Section  5 focuses on the interpretability of the models and explains the prediction decisions. Section  6 discusses the results, while Sect.  7 concludes the research with conclusions and future work.

## Background and related works

### Background

**Fig. 1:**
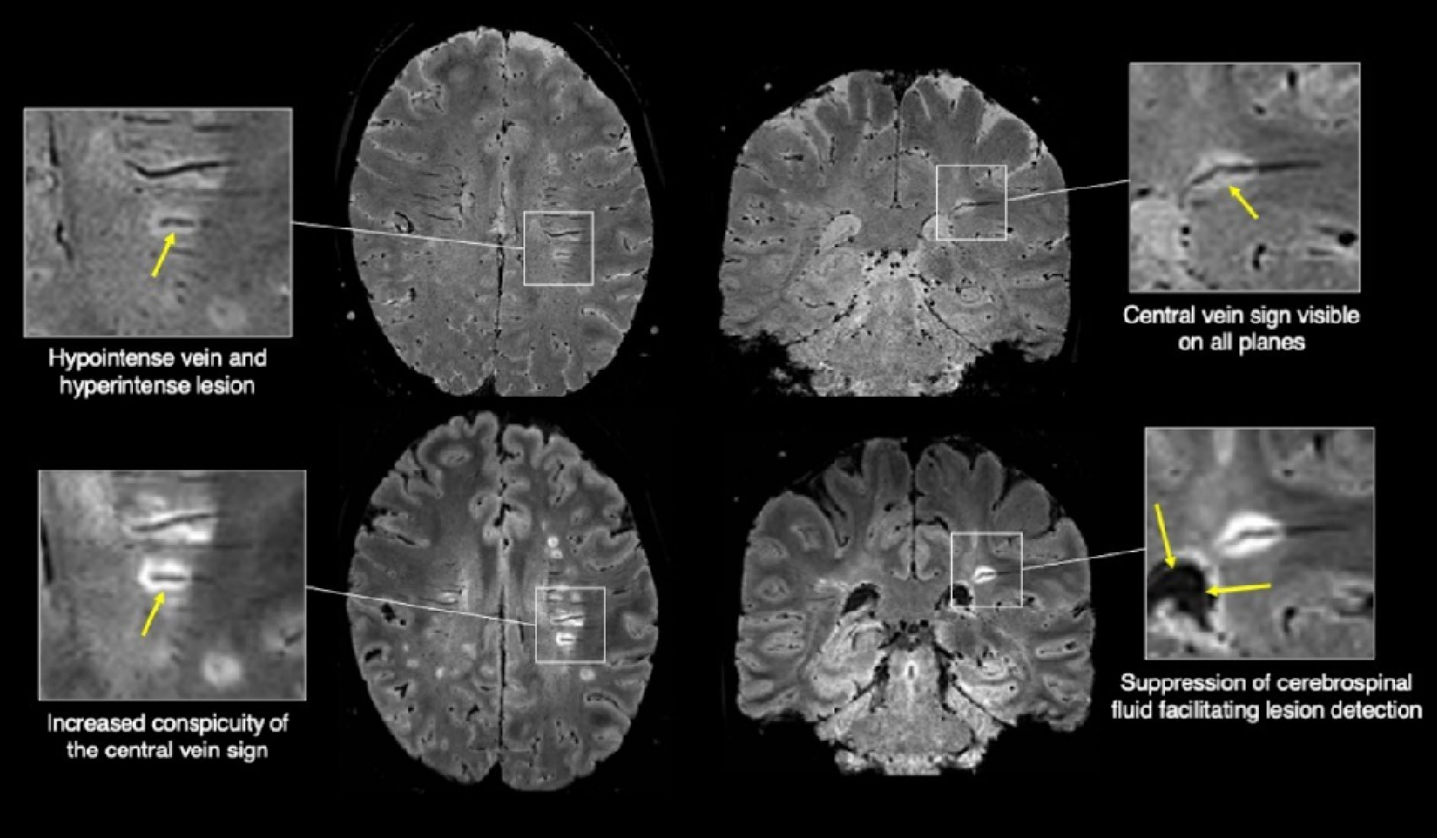
MRI technique for MS detection.

MS is an autoimmune disease that attacks the central nervous system. The myelin sheath surrounding the nerves begins to deteriorate, causing the nerves to lose their ability to transmit nerve signals properly, resulting in a decline in quality of life^12^. Therefore, MS patients require ongoing care and monitoring from a team of neurologists specializing in MS treatment^13^. However, doctors’ follow-up is sometimes inaccurate due to the low incidence of MS, resulting in limited diagnostic experience. Furthermore, because some symptoms resemble those of other diseases, relying on AI models to aid in diagnosis has proven valuable and has positively impacted treatment decisions^14^ (see Fig. 1).

**Fig. 2:**
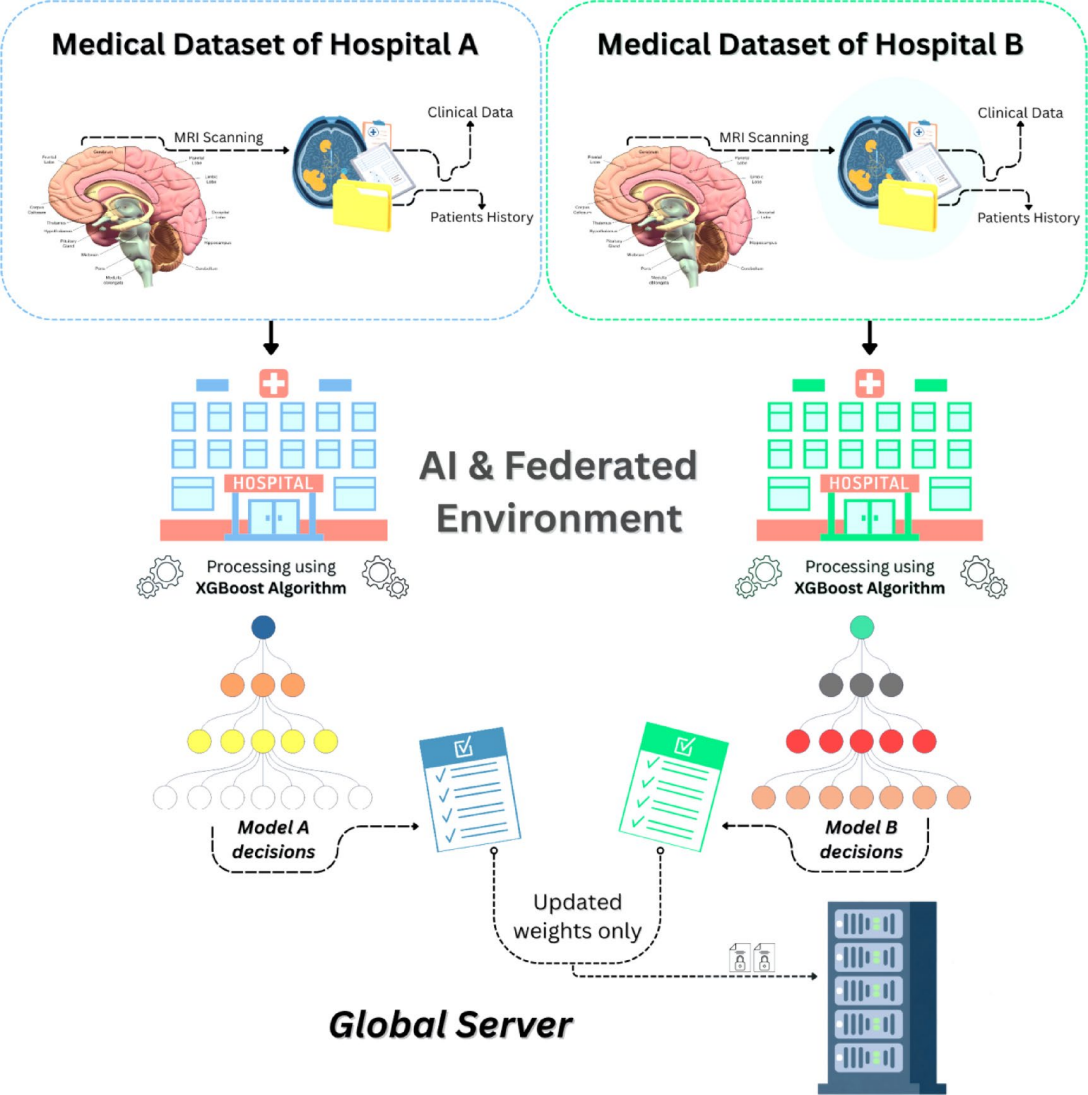
Sample of the processing of AI and FL models with MS.

The healthcare sector now relies heavily on electronic patient health records. Although these records contain highly confidential data, they are transferred from one system to another, exposing them to the risk of cyberattacks, data theft, or alteration^15^. However, FL has recently emerged as a suitable solution for maintaining the confidentiality of medical data, it’s a technique that focuses on improving data security and maintaining it during the aggregation of decentralized models, it doesn’t allow data to move between devices, but rather trains the model on each device with the data it contains, and the global server only takes the results of new developments^16^, as shown in Fig. 2 that represent sample of the processing of AI and FL models with MS, so it is now being used with the most serious diseases where data confidentiality is as critical as the treatment plan, such as cancer^17^.

### Related works

In a previous study by Rasouli et al.^18^ attempted to model disease progression by developing an interpretable machine learning approach that uses demographic, clinical, and MRI data to estimate the risk of transition from clinically isolated syndrome (CIS) to clinically definite multiple sclerosis (CDMS), the model was trained in traditional way on the same data that used in this paper from patients with CIS, and missing values were handled using multiple imputation with chained equations to maintain data integrity, and using an XGBoost for classification, the model achieved 83.6% accuracy during training and 78.3% during testing, and applied SHAP to explain model decisions to increase transparency and support clinical interpretation of disease progression.

In our previous study^19^, we compared the performance of two machine learning models on the same dataset that was used in this study, using Random Forest and XGBoost, and the latter proved to be the best, with an accuracy rate of 72% for Random Forest and 74% for XGBoost. Therefore, in this research, we relied on XGBoost and developed it to obtain the highest possible accuracy.

Moreover, Haggag et al.^20^ introduced a two-stage architecture for analyzing MS-related MRI data according to the increasing role of imaging in MS diagnosis. To more effectively capture subtle lesion patterns, we adopted Inception V3, pre-trained on RadImageNet, and refined its architecture with dimensionality reduction, dropout regularization, and Bayesian hyperparameter optimization, achieving high search performance with an average accuracy above 94% in various experimental settings.

Khattap et al.^21^ proposed a hybrid AI model combining deep learning and optimization algorithms, focusing on early prediction and disease progression assessment, to address data inflation and enable rapid MS progression prediction using T2-weighted MRI images. They used ResNet50 and multi-view ResNet architectures to inspect lesions from multiple perspectives and integrated view-space and view-channel attention blocks to focus on main spatial features and channel relationships. Feature selection was performed using the Quantum RIME algorithm, and the final classification using K-nearest neighbors achieved an accuracy of 98.29%.

In related research focusing on early lesion activity detection, Amini et al.^22^ developed an AI-based approach to identify active MS lesions without contrast injections due to concerns about contrast accumulation and potential health risks using T2-FLAIR MRI data from 130 patients with manually labeled active and inactive lesions. Several deep learning architectures were evaluated. EfficientNetV2M achieved the best performance, reporting an accuracy of 88% and an AUC of 0.92, while ensemble learning with adaptive weighting improved the robustness of the model.

To overcome privacy and data sharing constraints in multi-center collaborative studies, Bai et al.^8^ investigated federated learning for MS lesion segmentation, enabling collaborative model training across hospitals without the need to exchange raw MRI data. Using a robust label correction strategy based on a stronger global model to address discrepancies in lesion annotation, experiments on several multi-institutional datasets demonstrated that federated learning can achieve reliable segmentation performance while maintaining strict data confidentiality, even when annotation quality varies.

Zhang et al.^23^ observed that the first MRI scan performed on a CIS patient plays a significant role in accurate diagnosis, as it reveals the possibility of progression to MS. Therefore, they collected data from 84 patients with CIS and analyzed and segmented it using two methods: the first was traditional manual segmentation, and the second was using the LST tool, a fully automated tool. For classification, they used Oblique Random Forest, and the model achieved an accuracy of 84.5%, while the accuracy of traditional methods such as the McDonald criteria was 79%, so, in this research, we had to maintain the number of datasets used by imputing techniques for missing values, not dropped them, to ensure accurate learning and robust model training, as proven by the interpretation results when they matched the MacDonald criteria.

Xu et al.^24^ developed a model that differentiates between MS and cerebral small vessel disease, as there is a great similarity between the two diseases in MRI scans, which may lead to a misdiagnosis that leads to a deterioration in the patient’s health condition. Therefore, they built an AI model that helps to accurately differentiate between the two diseases using FLAIR-type MRI images; however, their study relied on differentiating between two existing diseases, which could delay the correct treatment plan for patients. In Table 1, there is a summary of Related Works.

**Table 1:** The summarization of related works.

Ref.	Dataset	Main objective	Challenges	Methodology	Year
^8^	Multi-institutional MRI datasets from several hospitals	To enable privacy-preserving collaborative learning for MS lesion segmentation without sharing raw medical data	The study addresses challenges related to data privacy constraints, inter-hospital data heterogeneity, and inconsistencies in lesion annotations across institutions.	The proposed approach applies federated learning for distributed model training combined with a robust label correction strategy guided by a stronger global model to mitigate annotation noise and improve segmentation reliability.	2024
^18^	273 CIS patients	Develop a transparent and interpretable ML model that predicts the risk of CIS converting to CDMS.	Loss of a large amount of data and long-term follow-up, which may cause patient absence or loss of their data, and a gap in the efficiency of the model between training and testing.	To solve the data loss problem, MICE was used, and for the prediction process, XGBoost was used, where the accuracy was 78.3% during testing, and for interpretation, SHAP was used.	2024
^19^	273 CIS patients	Predict the conversion from CIS to MS and improve early diagnosis using an optimized machine learning model.	The study addresses challenges related to missing clinical data, limited dataset size, class imbalance inherent in CIS-to-MS progression cases, and the difficulty of accurate early diagnosis due to disease complexity and variability.	The approach applies MICE imputation for missing data handling, feature engineering for improved representation, and an optimized XGBoost classifier tuned via hyperparameter optimization. Performance is compared against Random Forest to validate improvements.	2026
^20^	Heterogeneous workplace survey datasets + external occupational burnout dataset	Develop a calibrated, interpretable ML framework for occupational mental-health risk prediction and subgroup profiling.	heterogeneous survey data, the need for well-calibrated probabilistic predictions, limited model interpretability, fairness across different subgroups, and performance degradation under dataset shift and cross-domain settings	The proposed approach integrates attention-based deep learning for tabular data, variational autoencoders for latent behavioral profiling, ensemble learning for robust prediction, probability calibration techniques, feature attribution methods for interpretability, and cross-dataset evaluation to assess generalization.	2025
^21^	425 MRI images.	Develop a hybrid AI framework that combines ResNet with a swarm optimization algorithm for accurate early diagnosis of MS from MRI.	The small amount of data increases the risk of overfitting and requires devices with powerful processing capabilities to process complex images, as the features in them have high dimensions.	ResNet was used for feature extraction from MRI, and Swarm Optimization algorithms. The KNN algorithm was used to classify people with MS from healthy people, achieving an accuracy of 98.29%.	2025
^22^	9097 lesion images from 130 MS patients.	Classify active vs. inactive MS lesions using only non-contrast FLAIR MRI, as an alternative to gadolinium-based contrast agents	Manual ROI selection is required, which may lead to the unintentional loss of a pest.	They used a custom CNN and compared it with 12 transfer learning CNNs (ResNet50, VGG16/19, etc.). The best result was Ensemble learning with CNN + ResNet50 + VGG19, with an accuracy ≈ 95%	2024
^23^	84 CIS patient MRI scans	Improve early diagnosis of MS progression from CIS using MRI-based analysis and classification	Small dataset size, missing data handling, variability between manual and automated segmentation, and need for reliable interpretation aligned with clinical criteria (McDonald criteria)	MRI preprocessing + feature extraction via manual segmentation and LST tool, missing value imputation, classification using Oblique Random Forest, performance comparison with clinical baseline methods	2019
^24^	FLAIR MRI scans distinguishing MS patients and cerebral small vessel disease cases	Develop a model to differentiate MS from similar neurological diseases to reduce misdiagnosis risk	High visual similarity between MS and small vessel disease lesions, risk of misdiagnosis, and potential delay in correct treatment	AI-based classification model using FLAIR MRI features for binary disease discrimination (MS vs. non-MS/CSVD)	2024

## Proposed methodology

### Model training

Extreme Gradient Boosting was used in the prediction process because the algorithm relies on Sequential Learning^25^. This means it’s not simply a series of trees carrying decisions^26^; each tree attempts to correct errors in previous trees. It also incorporates built-in regularization^27^, which, in our case (where the model deals with a small amount of data), proved highly effective in preventing overfitting.

In the ML model to maximize the algorithm’s effectiveness, an Automated Model Tuning Hyperparameter was implemented using Grid Search Cross-Validation. The system automatically tested and evaluated all possible combinations using 5-Fold Cross-Validation. This methodology ensures complete stability and accuracy from the model itself, without human intervention, thus increasing the reliability of the results.

**Fig. 3:**
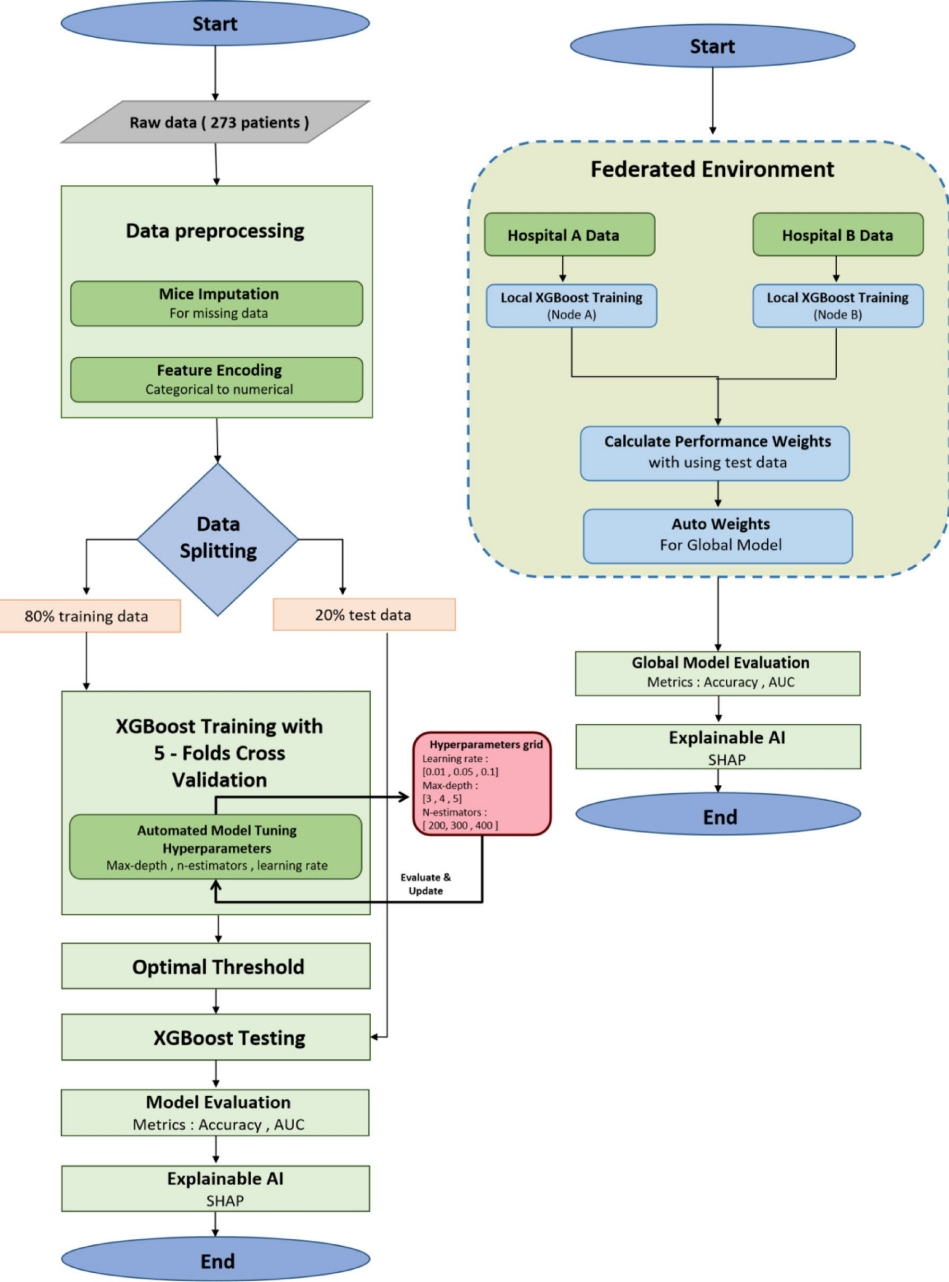
Proposed methodology for XGBoost model, integrating automated model tuning hyperparameter optimization, and in-silico simulation of FL model.

In the federated model, each hospital was trained only on its own existing data. For example, the XGBoost model at Hospital A learned only the data patterns of available patients. This helped prevent data leakage and maintain confidentiality. Auto-Weighted Voting further increases the model’s advantage, as some hospitals might have significantly more patient data than healthy data, potentially leading to overfitting in their results. Therefore, the model with the most accurate results is given a higher weight, and only software updates are sent to the central server. Figure 3 illustrates the proposed research methodology, outlining the training path for both the ML model using the XGBoost algorithm and the Manual FL model. The diagram highlights how intelligent data processing is integrated with automated tuning mechanisms, culminating in the final evaluation and interpretation of results.

### Model and hyperparameters

This study presents an advanced early prediction model for MS that achieves a balance between high computational metrics and the highest data security standards. The XGBoost algorithm was used in the prediction process, supported by cross-validation to prevent overfitting, and a simulation model for FL was employed to ensure the confidentiality of medical data.

ML algorithms were used due to the small dataset. XGBoost, a supervised learning algorithm, was employed, which is suitable for data in row and column formats like CSV, the format used in our research. Automated model tuning was used in the training process, so the model performed 30 iterations of the data with different hyperparameter values to determine the optimal configuration. However, with cross-validation in the training process and the data being divided into 5 folds, the model effectively performed 150 iterations, optimising its experience without the risk of overfitting. Therefore, the training data was divided into five folds to prevent overfitting. The model was trained on four folds at a time and tested on the fifth using different hyperparameters until the optimal hyperparameter was identified. The best hyperparameters were defined as shown in Table 2.

**Table 2:** Best hyperparameters for ML Model.

Hyperparameter	Value
Subsample	0.7
N estimators	400
Max depth	5
Learning rate	0.1

When implementing the federated model, multiple hyperparameters were manually tested to select the best one. This prevented the federated model from becoming more complex or consuming more resources, and also avoided the risk of overfitting, as each device in a federated model works on a smaller amount of data.

### Statistical analysis

Statistics were compiled on the basic clinical and demographic characteristics of the patients participating in the study, dividing them into two groups: those whose condition remained stable (Non-MS) and those whose condition progressed to MS cases, as shown in Table 3. Regarding age, the average age of those affected was 33, confirming medical findings indicating that the disease is more prevalent in younger age groups^28^. As for gender, the percentage of affected females exceeded 51%, which supports medical theories^29^. The percentages of positive OCB and peripheral lesions were 47.2% and 80.0%, respectively, which are two of the most significant indicators. This supports the McDonald theory, which suggests that the presence of periventricular lesions is a major risk factor for MS^30^. All these statistics were obtained using the T-test and Chi-Square in Python and its libraries such as Pandas.

**Table 3:** Clinical and demographic characteristics of the patients participating in the study.

Clinical feature	MS cases (*n* = 148)	Non-MS (*n* = 125)	*P*-Value	Statistical test	Clinical significance
Age (mean ± SD)	33.41 ± 10.85	34.84 ± 11.42	0.2888	T-Test	NS (Not Sig.)
Schooling (mean ± SD)	14.45 ± 4.23	16.02 ± 4.10	0.0021	T-Test	** (Very Sig.)
Female gender (%)	64 (51.2%)	41 (27.7%)	0.0001	Chi-Square	*** (Highly Sig.)
History of breastfeeding (%)	65 (52.0%)	66 (44.6%)	0.5073	Chi-Square	NS (Not Sig.)
History of varicella (%)	60 (48.0%)	64 (43.2%)	0.5185	Chi-Square	NS (Not Sig.)
Positive OCB (%)	59 (47.2%)	17 (11.5%)	0.0000	Chi-Square	*** (Highly Sig.)
Polysymptomatic presentation (%)	32 (25.6%)	49 (33.1%)	0.3612	Chi-Square	NS (Not Sig.)
Periventricular lesions (%)	100 (80.0%)	38 (25.7%)	0.0000	Chi-Square	*** (Highly Sig.)
Cortical lesions (%)	69 (55.2%)	49 (33.1%)	0.0004	Chi-Square	*** (Highly Sig.)
Infratentorial lesions (%)	63 (50.4%)	17 (11.5%)	0.0000	Chi-Square	*** (Highly Sig.)
Spinal cord lesions (%)	47 (37.6%)	39 (26.4%)	0.0625	Chi-Square	NS (Not Sig.)
Abnormal VEP (%)	52 (41.6%)	32 (21.6%)	0.0006	Chi-Square	*** (Highly Sig.)
Abnormal BAEP (%)	10 (8.0%)	8 (5.4%)	0.5380	Chi-Square	NS (Not Sig.)
Abnormal LLSSEP (%)	68 (54.4%)	48 (32.4%)	0.0004	Chi-Square	*** (Highly Sig.)
Abnormal ULSSEP (%)	59 (47.2%)	42 (28.4%)	0.0020	Chi-Square	** (Very Sig.)

## Experimental results

The study included Mexican patients with CIS. The data included clinical, radiological, and demographic factors that helped determine whether a patient’s condition would progress to MS. Missing values in the dataset were replaced using iterative imputers applying MICE to preserve the influence of features on each other. The XGBoost model was used for classification and to distinguish between patients with and without MS. For explanation, the model’s decision SHAP technique was used, and to maintain data confidentiality, in-silico simulation of FL was applied.

To measure the model’s stability, cross-validation was used. This is the scientific method that ensures the model did not memorize data and that the accuracy and AUC it achieved are a result of learning, not overfitting, to confirm the model’s accuracy and sensitivity, global metrics, accuracy and ROC-AUC, were used and The step that logically and scientifically confirmed the model’s results was the SHAP, when the model identified Periventricular MRI and MRI Lesion Count as its most important features, it aligned with the international medical standards McDonald Criteria.

The ML and in-silico simulation of FL models were conducted on a 64-bit desktop system equipped with an Intel Core i7-6600U processor operating at 2.60 GHz, 8 GB of RAM, and a 256 GB solid-state drive (SSD), and designed using Python with Scikit-Learn for StratifiedKFold, IterativeImputer, and library XGBClassifier. Explanatory Calculations: SHAP library for local and global calculations. Graphing and Schematics: NumPy, Pandas, Matplotlib, and others.

### Dataset description

The study was conducted on 273 Mexican patients who were newly diagnosed with CIS. This study was conducted at the National Institute of Neurology and Neurosurgery (NINN) in Mexico City between 2006 and 2010. Although the size of data presents a significant challenge, it can be used to improve predictive performance, demonstrating the importance of effective knowledge transfer and model generalization under data-constrained conditions^31,32^. The dataset includes comprehensive medical and demographic information, including: Demographics: age, years of schooling, and sex. Medical history: breastfeeding and a history of chicken pox. Clinical symptoms: type of primary symptoms (visual, motor, sensory, etc.) and whether there were single or multiple symptoms. Specialized neurological examinations: Evoked potentials (EPs), such as visual acuity tests (VEP), auditory evoked potential tests (BAEP), and sensory evoked potential tests (SSEP). Cerebrospinal fluid analysis: detection of oligoclonal bands. Magnetic resonance imaging (MRI): imaging results from various regions, such as the cerebral cortex and spinal cord. Disability scales (EDSS): a scale for assessing the level of neurological disability at onset and onset^33^. The data was obtained from the Mendeley database, where it is available by^34^.

All computational and analytical methods in the current study were performed in accordance with the relevant guidelines and regulations. The original database was approved by the NINN Institutional Review Board (Approval No. 28/11), and written informed consent was obtained from all participants, as stated by Víctor Chavarria et al.^35^. Therefore, when dealing with the data in this study, no additional institutional ethical approval was required.

### Data pre-processing

This dataset contains 273 patients. The data was divided into 20% testing (55 patients) and 80% training (218 patients). Cross-validation was used in the training process. In the RandomizedSearchCV, a 5-fold configuration was used, and the subsample data was 70%, leaving 174 patients for training and 30% (44 patients) for validation. This was done to prevent overfitting. In the federated data, 50% was divided between the two hospitals, so that each hospital had 109 patients.

Most of the columns in the data containing the number 3 indicated unknown data. So, we started selecting the number 3 in these columns and assigning it `nan`. After that, we confirmed that all the features we had had complete data, or at least that no more than 50% of the data was missing. If a column had a missing percentage greater than 50%, we deleted it. However, this wasn’t the appropriate method to use for the remaining missing data, so we used the MICE method^36^ because it replaces missing values with values resulting from statistical analysis that don’t affect the correlation coefficient between the data, to further ensure data leakage, any missing values were compensated for after the data division, and the testing was conducted using the prepared data.

Making a feature engineer to add two extra features. The first is MRI_Lesion_Count, which is responsible for identifying and compiling all the affected areas in the brain as a result of MRI scans. The second is Symptom_Count, which counts the symptoms that appear to indicate the severity of the disease^18^. That means if someone’s Symptom_Count = 3, they have three symptoms, and therefore their condition is more serious than someone who only has one symptom. Feature Engineering was performed on an individual basis, completely independent of group statistics, thus maintaining data isolation throughout the validation phases.

### Metrics results

The efficiency of the ML model was measured using cross-validation and multi-metric techniques to confirm the model’s results. The ML model utilized automated model tuning and robust validation implemented using randomized search cross-validation. Since the data used in each round ranged between 54 and 55, due to the data was divided into 5 folds. This technique ensures fair distribution of data, with an equal proportion of sick and healthy individuals in each fold. The results shown in Table 4, the results using multi-metric techniques, were: Accuracy = 81.8%, ROC-AUC = 88%, and Recall = 76.9%, and we have calculated the 95% Confidence Intervals (CI), which demonstrate the high stability of the model, as the confidence interval is narrow, ranging between 87.13% and 93.67%. This statistically proves that the model operates with consistent and near-certain efficiency even if the patient samples change. We were also able to apply a confusion matrix based on mathematical equations in (1–5) to extract the values of True Positives (TP), True Negatives (TN), False Positives (FP), and False Negatives (FN)^37,38^.

1 $$\:Accuracy\:=\:(TP\:+\:TN)\:/\:(TP\:+\:TN\:+\:FP\:+\:FN)$$

2 $$\:Recall\:=\:TP\:/\:(TP\:+\:FN)$$

3 *Precision=TP / (TP + FP)*

4 *Specificity=TN/(TN+FP)*

5 $$F1-Score=2\times (Precision\times Recall)/(Precision + Recall)$$

However, the efficiency of the federated model was measured using multi-metric techniques only. The model achieved an accuracy of 76.3%, meaning it correctly diagnosed 76 out of every 100 cases, and a recall rate of 80.7%, meaning it detected approximately 81% of individuals who progressed to MS. It also achieved an ROC_AUC of 83.9%, reflecting a high predictive capability.

**Table 4:** Results of cross validation on ML model final test.

Metric	Test	Standard deviation	95% CI
Accuracy	80.2%	0.0445	[71.48%, 88.92%]
AUC	90.4%	0.0167	[87.13%, 93.67%]

The model demonstrated high accuracy and ROC-AUC. Accuracy alone was not sufficient, as the ability to differentiate between patients and healthy individuals is crucial in medical data, so ROC-AUC was also considered, demonstrating the model’s high performance, successfully differentiating between the most affected cases in the transition from a first attack to MS. The training accuracy was 96.7%, the ROC-AUC was 99%, and the validation result was 92.5%.

**Fig. 4:**
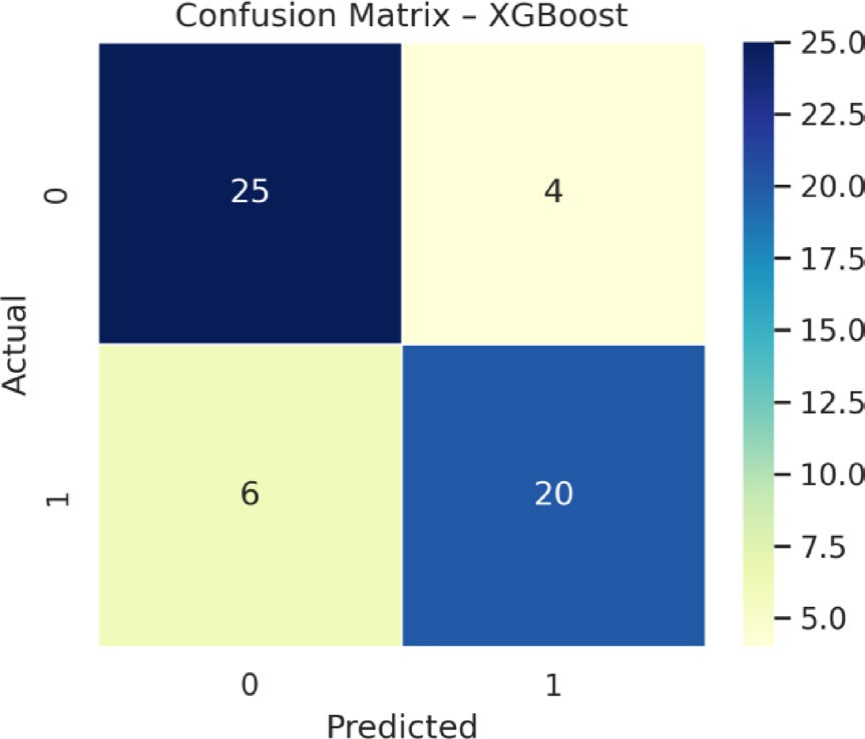
Confusion matrix of the results of ML model before applying federated simulation.

To make the ML model more customized, the model defines its optimal threshold to 0.72 so that the model can determine the probability of the person being infected at any given value. When the model was tested, its accuracy was 81.8%, ROC-AUC was 88%, and sensitivity was 76.9%. The model’s performance in classifying cases that convert from CIS to MS and those that remain in CIS is shown by the Confusion Matrix shown in Fig. 4, which leads to more confident clinical decision-making. According to the outcomes derived from Fig. 4, additional clinically relevant evaluation metrics—including Precision, Specificity, and F1-Score were calculated using Eqs. (3), (4), and (5) to provide a deeper statistical validation. These comprehensive final test results are summarized in Table 5, that shown the final test evaluation metrics for the Proposed ML Model, and by applying Matthews Correlation Coefficient (MCC), we find that achieve 60.1% which, confirming a robust, well-above-chance predictive power.

**Table 5:** Final test evaluation metrics for the proposed ML model (threshold = 0.72).

Evaluation metric	Final test value
Accuracy	81.8%
ROC-AUC	88.0%
Recall (sensitivity)	76.9%
Precision	83.3%
Specificity	86.2%
F1-Score	80.0%

**Fig. 5:**
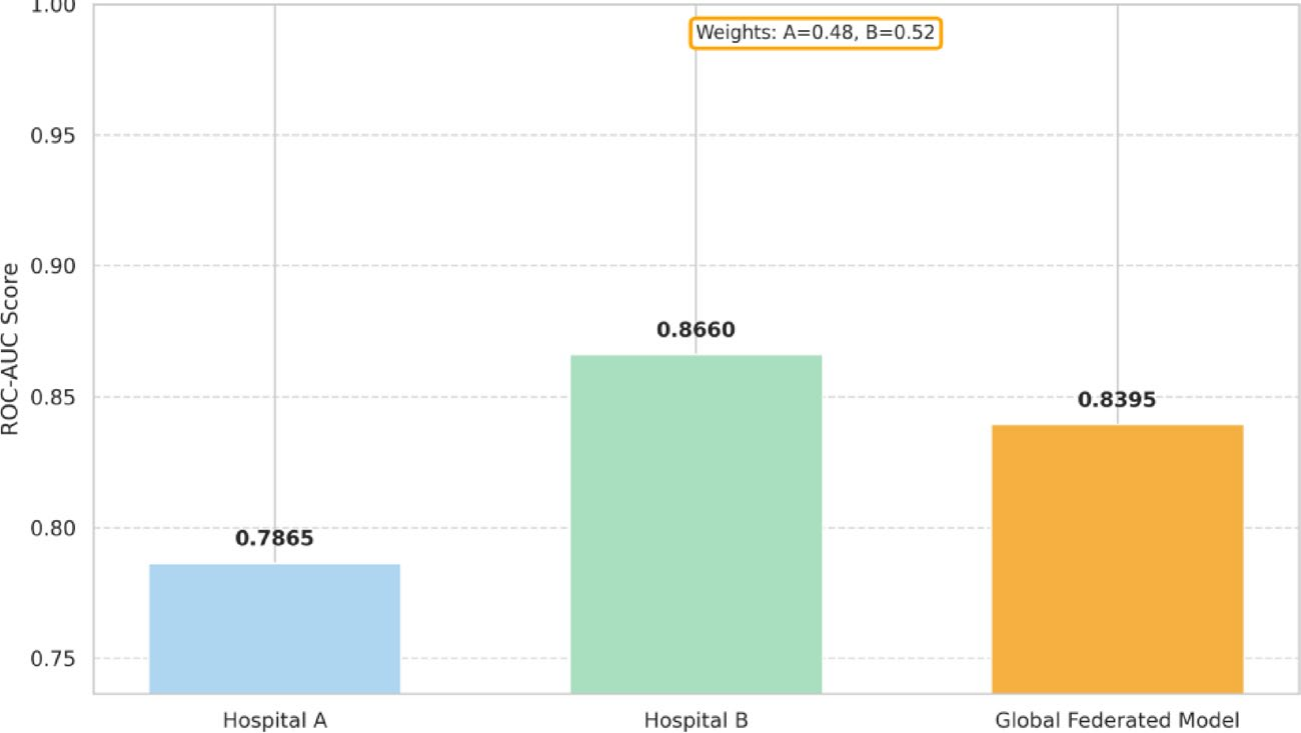
Comparison between local models (Hospital A, Hospital B) and global federated model.

The federated model achieved high performance in receiving and combining the classification results of each model from different locations while maintaining high predictive accuracy. The data was divided equally between two local models, and the results from each were used to process the global model. The results for each model were: Hospital A (Local model) accuracy = 74.5% and AUC = 78.6%, Hospital B (Local model) accuracy = 72.7%, AUC = 86.6%, where the global model achieved an accuracy = 76.3% and an AUC = 83.9%, as shown in Fig. 5.

Despite relying on the same database as Rasouli^18^, the model in this research achieved higher accuracy when using automated model tuning and robust validation implemented using randomized search cross-validation in the training process. Instead of training the model on the entire dataset, it divided it into five folds and trained on them in parts. This was done using the best hyperparameter, which the model identified after training a portion of the training data. The entire training data was then trained based on this hyperparameter. Table 6 compares the values of the machine learning model methods implemented by Rasouli and those implemented in this study.

**Table 6:** Results of multi-metric in Rasouli experiment and our experiment.

Reference	Accuracy	ROC-AUC
^18^	78.3%	85.8%
Proposed model	81.8%	88%

## Model interpretability and explainable AI (XAI)

To interpret the model results, the 15 most influential features were extracted using two methods: (1) using the feature importance feature provided by XGBoost, (2) using the KernelExplainer from SHAP to rank feature values and observe how the output changes, and the SHAP Summary Plot was recently used to visualize the directionality of their effects^39,40^. Both methods are used in both ML model and federated model.

### Features importance

**Fig. 6:**
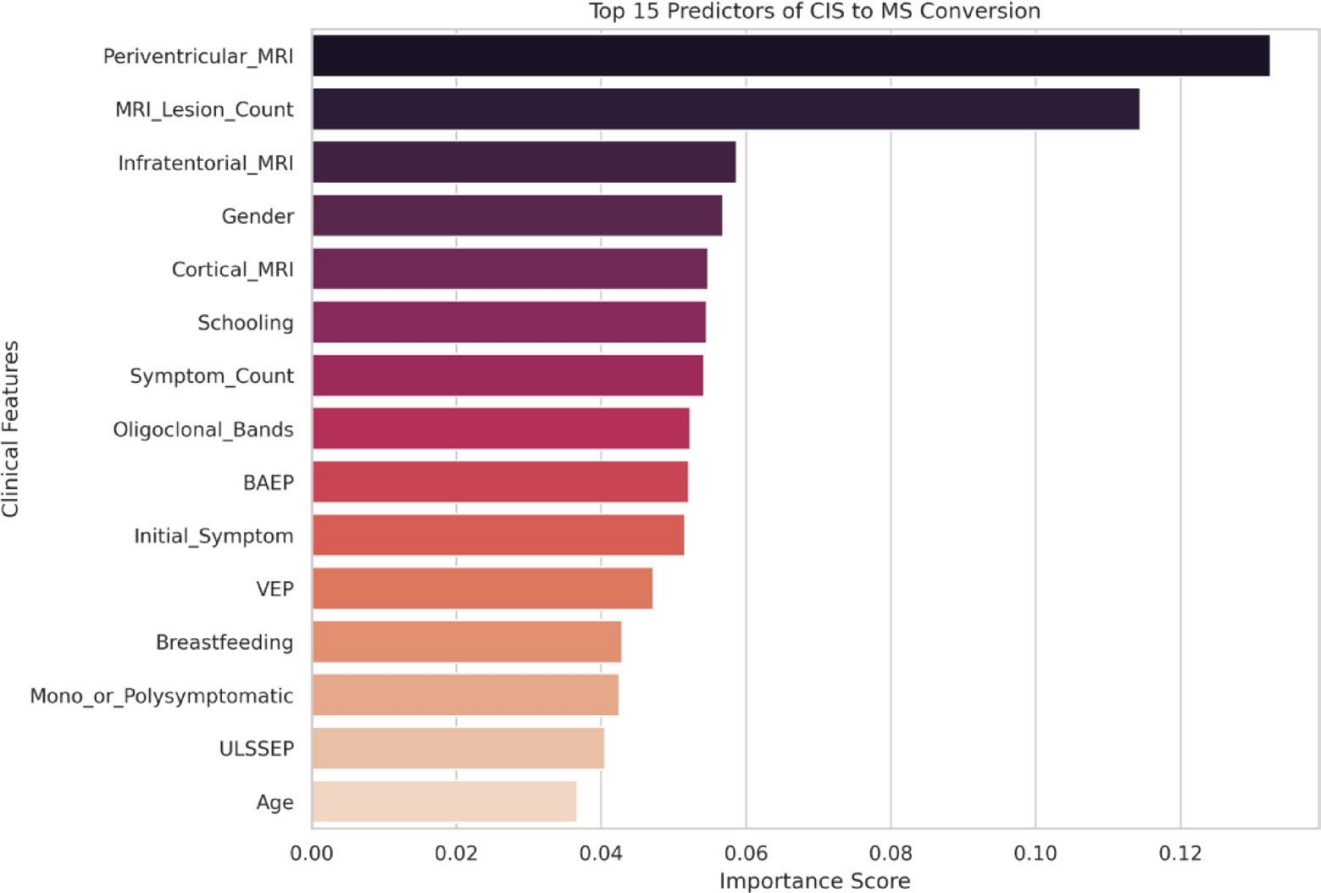
The result of feature importance for ML model before applying federated simulation.

**Fig. 7:**
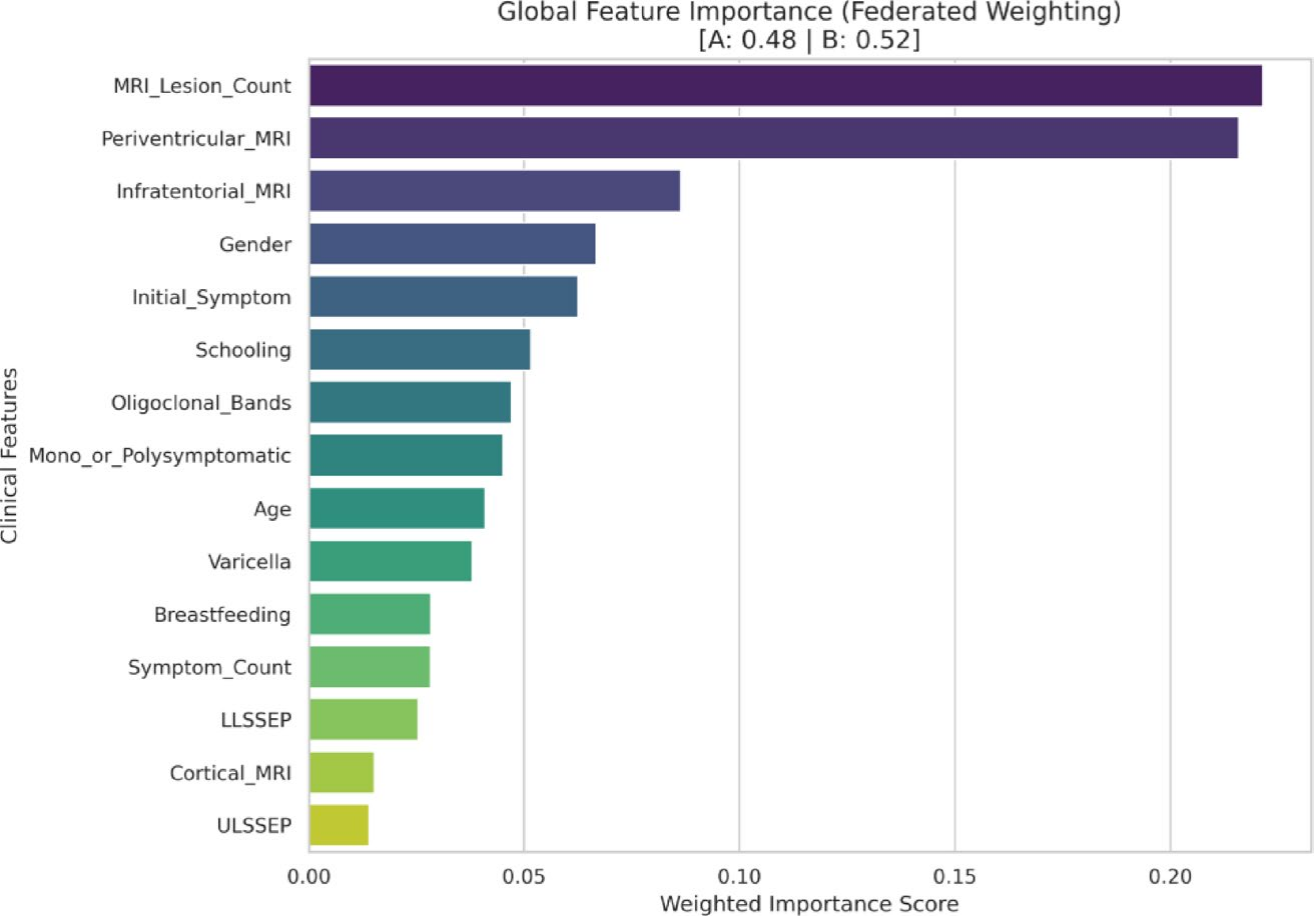
The result of feature importance for federated model.

**Fig. 8:**
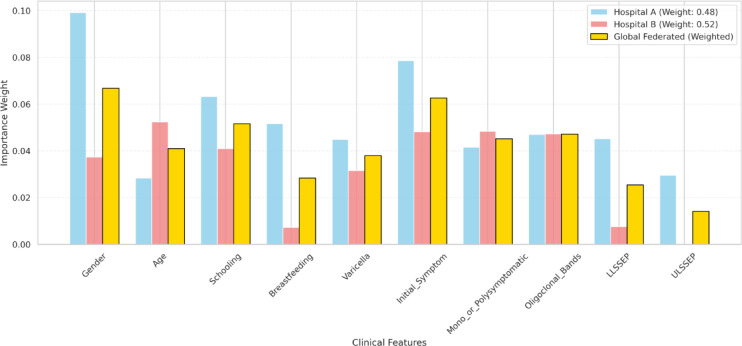
Feature importance alignment: local vs. global federated model.

We find that the ranking of the most 4 effective clinical features in Fig. 6, which represents the ML model, gives the same results as in Fig. 7, which represents the most effective features on the federated model, this indicates that the federated model was able to learn well even after splitting the data and taking only the results of the two models, the difference in their ranking is due to a gap between the results of the first and second local models for the Federated application, which affected the global model when they were combined. In Fig. 8, we will see the extent of each feature’s effect on model training with respect to federated models.

### XAI

AI alone provides high accuracy and sound, logical decisions, but without explaining the reasoning behind those decisions. Therefore, using XAI was crucial in interpreting the results to build trust between the model and the user, and recent healthcare studies have presented the role of interpretable and calibrated predictive models in supporting reliable clinical decision-making^41^. For this reason, we used SHAP, which relies on a mathematical theory called Shapley Values, assigning each feature a percentage based on its impact on the results.

**Fig. 9:**
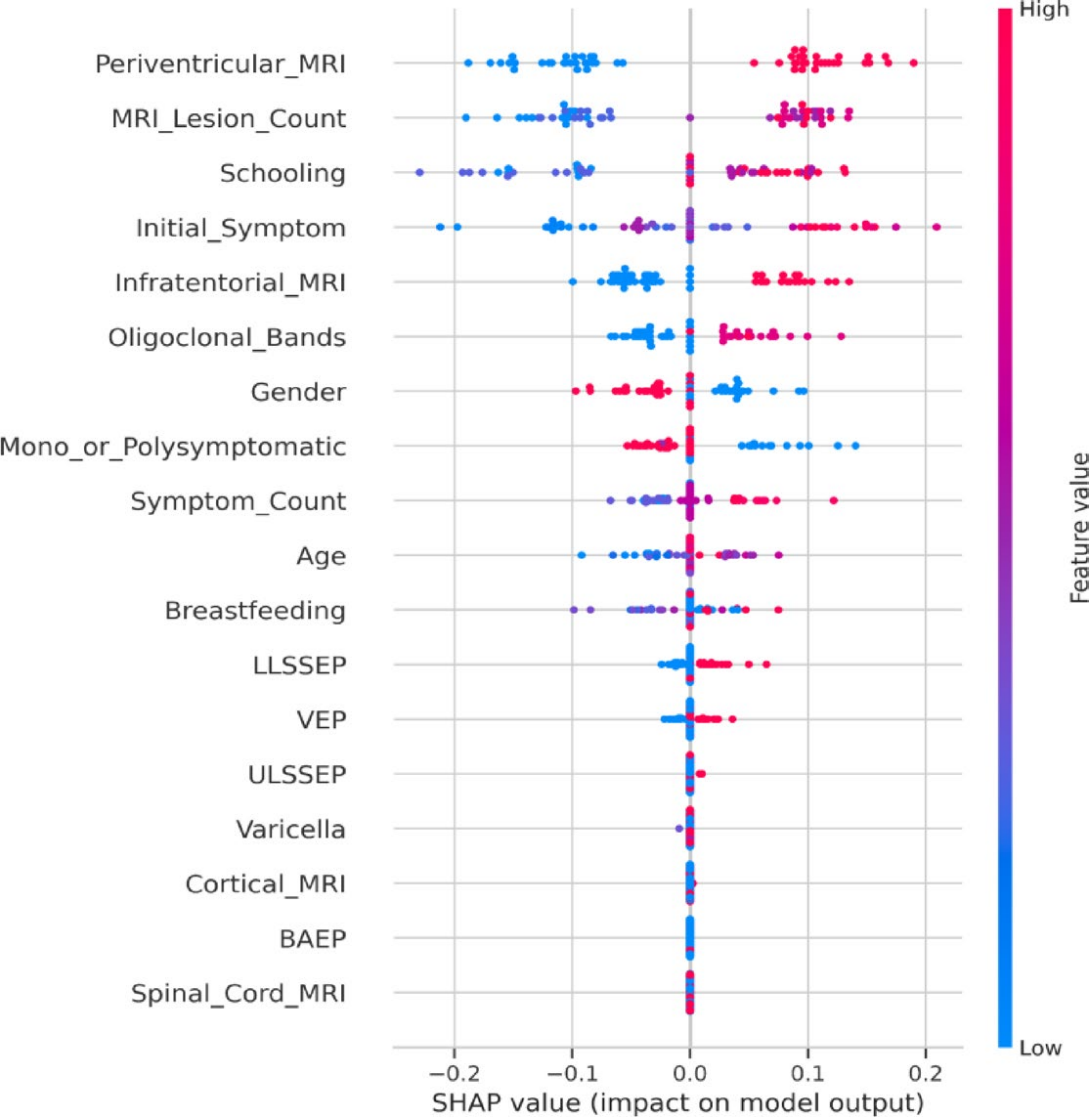
SHAP summary plot for ML and federated models, as both are the same.

**Fig. 10:**
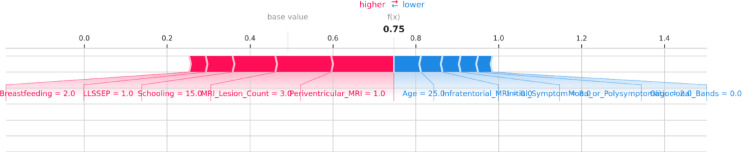
SHAP Force Plots for Individual Patient Predictions for ML model, which shows how different features contribute to the final decision; the presence of MRI lesions raised the probability of conversion to 75%, exceeding the threshold required for diagnosis.

In both the ML model and the federated model, we used feature importance to extract the most important features that helped the model achieve high efficiency and to exclude weak features that had no impact. Then we used SHAP to interpret the model results, and both yielded the same results, as shown in Fig. 9, where The vertical order from top to bottom represents the order of variables from most important to least important, while the horizontal order represents the range of points that represent the extent of the variable’s influence on changing the model’s decision, where red represents a high value for the variable and blue represents a low value, and points on the right push the model to predict MS Conversion and points on the left push the model to predict Non-MS^42^, and Fig. 10 for individual case in ML model.

We will find that the most influential features on the model’s final decision are Periventricular MRI and MRI Lesion Count. This demonstrates the model’s high diagnostic efficiency, as, according to medical standards, the more lesions around the ventricles, the higher the probability of developing MS. It is also established that the more widespread the scarring and the greater the number of affected areas, the higher the likelihood of the patient experiencing multiple attacks that could lead to MS, as shown in Fig. 9, the presence or absence of MRI features Periventricular MRI and MRI Lesion Count has the greatest impact on the model’s decision, with average effect values ranging from − 0.18 to + 0.15 and from − 0.15 to + 0.12, as detailed in Table 7.

This supports the McDonald Criteria this criteria relies on two fundamental pillars of diagnosis: Dissemination in Space (DIS), which focuses on the areas of disease spread, as this determines the severity of the patient’s condition, and Dissemination in Time (DIT), which focuses on the time period during which the disease spread to indicate whether the patient’s condition is stable or worsening, where the presence of periventricular scarring is a key medical criterion for proving DIS. Figure 10 shows that the distribution of MRI features demonstrates a positive correlation between this feature and disease progression. Clinically, according to the McDonald Criteria, a high number of lesions in the periventricular region (high redness) elevates the SHAP value above zero, providing strong clinical evidence for disease dissemination and thus accurately predicting progression to MS. Its absence, represented by the blue color, pushes the points to the left as a protective factor that keeps the patient in a stable CIS state. As for age, this illustrates the Inverse Relationship, since medically, the appearance of isolated neurological symptoms at an early age is a serious clinical indicator of aggressive disease activity. Thus, the model picked up this clever pattern; it found that young age, represented by the blue color, increases the likelihood of transformation to MS, which is represented by the right of the line, while infection at a later age, represented by the red color, may mean a slower course of the disease, which is shown on the left of the line.

A quantitative comparison of the SHAP summary measures was carried out in order to verify the interpretational congruence between the two frameworks mathematically. The central XGBoost model and the unified global model both retained the same global importance hierarchy for the 11 important clinical variables, as Table 7 illustrates. Excellent structural similarity was demonstrated by the precise mathematical bounds of SHAP effect ranges for main diagnostic indicators, such as periventricular MRI status and total number of MRI lesions.

**Table 7:** Quantitative alignment of SHAP interpretation between centralized and federated models.

Feature ranking	Centralized XGBoost	Federated global model
1st	Periventricular_MRI [− 0.18, + 0.15]	Periventricular_MRI [− 0.18, + 0.14]
2nd	MRI_Lesion_Count [− 0.15, + 0.12]	MRI_Lesion_Count [− 0.14, + 0.12]
3rd	Schooling [− 0.12, + 0.08]	Schooling [− 0.12, + 0.08]
4th	Initial_Symptom [− 0.10, + 0.14]	Initial_Symptom [− 0.10, + 0.13]
5th	Infratentorial_MRI [− 0.06, + 0.08]	Infratentorial_MRI [− 0.05, + 0.08]
6th	Oligoclonal_Bands [− 0.05, + 0.06]	Oligoclonal_Bands [− 0.05, + 0.05]

## Discussion

A predictive model that uses only ML can be a good aid in guiding the patient towards the right treatment path^43^. In our study we use ML models, it is more than DL as it needs large data or data that needs a specific mechanism, as in^44^, helping them control and manage the progression of the disease and appropriate drugs^45^. However, the greatest benefit is that this is done while maintaining the confidentiality of patient data and obtaining accurate predictive results.

Rasouli et al.^18^ The study relied on the same dataset used in this research, but we developed an ML model to achieve more accurate predictive results. They used XGBoost and achieved an accuracy of 78.3%. Therefore, we should have achieved even higher accuracy, as medical data is sensitive and any decision can be life-threatening for the patient.

In this study, we provided a model that predicts disease transformation before its progression to ensure control over attacks affecting patients while focusing on collecting data from multiple sources using simulation models for federated learning to increase the model’s learning ability and generalizability.

**Fig. 11:**
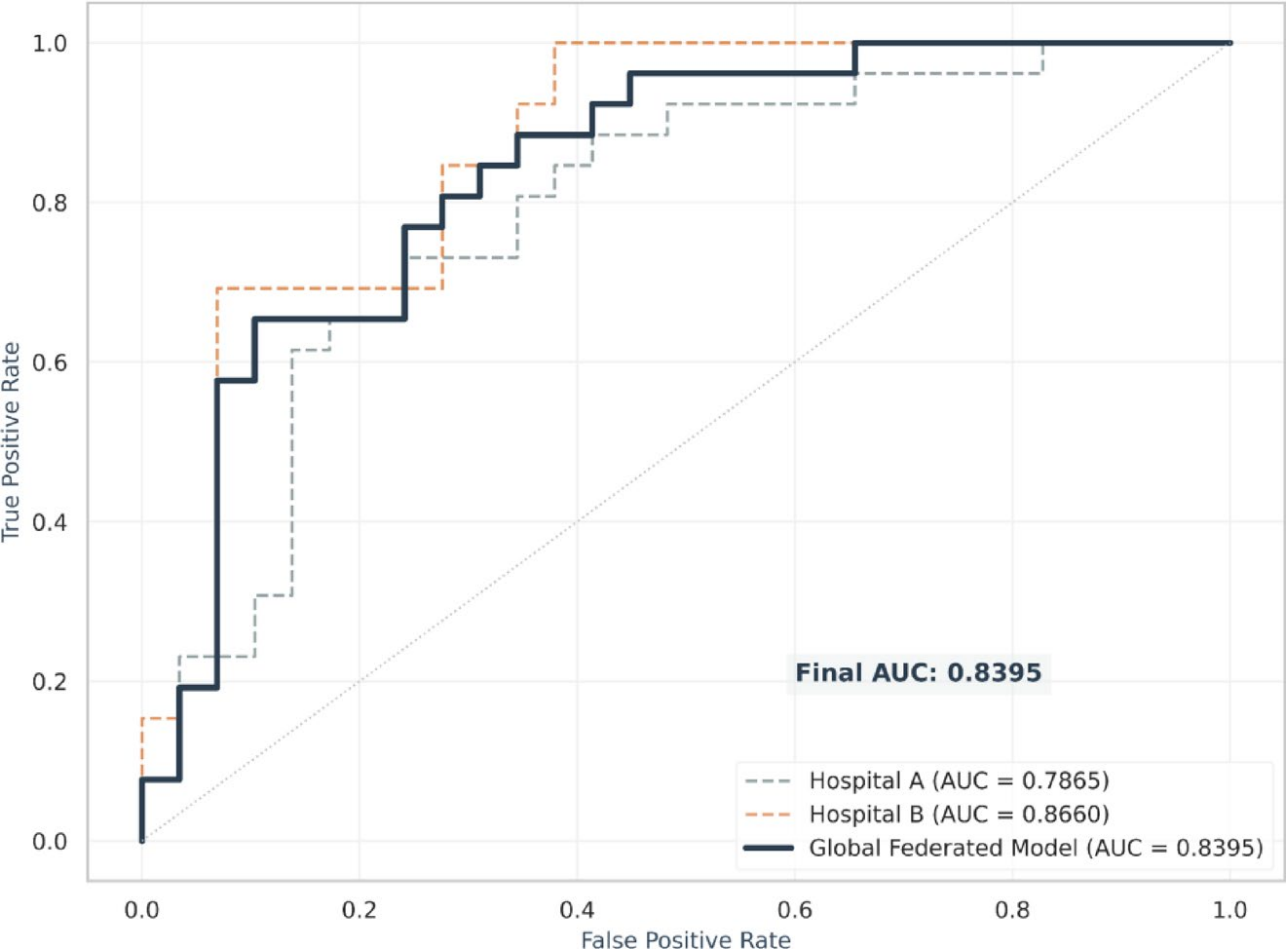
ROC-AUC between local models and global models.

In our study, we relied on feature engineering, that proved in recent studies it iis very important in ML models and utilizing all the existing features to increase accuracy to reach 81.8%, relying on important medical features such as Periventricular_MRI, which represent the most important factors for MS disease according to medical standards, and the increase in the number of affected areas in the patient that represents in MRI_Lesion_Count with the association of scar tissue with Infratentorial MRI, and the gender that proves the scientific theories indicating that females are more susceptible to infection. Recent developments in machine learning applications across different fields indicate a growing trend toward integrating interpretability, advanced feature learning, and efficient model optimization, which further supports the need for reliable and explainable AI systems in high-stakes healthcare prediction problems^46,47^. Due to the model’s high learning ability with these features and others, the federated model was able to achieve high efficiency when dividing the data into two local models and relying only on the results coming out of them. It showed the high ability of the global model to differentiate between a CIS patient and an MS patient, and this is what the global ROC-AUC results showed, which recorded 83.95%. Figure 11 illustrates the degree to which each local model differentiates between the two classes (MS and NonMS), the ability of the global model to differentiate, and the extent of their influence on it.

### Error analysis and clinical implications

A false negative occurs when a patient actually has multiple sclerosis, but the model predicts they are healthy. This often happens in the early stages of the disease or in cases where clinical symptoms are mild and atypical, as the biomarkers overlap with those of healthy individuals. This is the most serious diagnosis because it delays treatment and allows the disease to spread. Therefore, the Threshold was set at 0.72 to minimize these cases as much as possible to ensure patient safety. In our study, there were only 6 false negatives, and the patients were carriers of the disease, demonstrating the model’s effectiveness.

False positives occur when a person is healthy or has symptoms similar to those of another disease, yet the model predicts multiple sclerosis (MS). This happens because MS presents with similar clinical and diagnostic findings to other neurological and autoimmune diseases. This statistical similarity in the data causes XGBoost’s decision trees to classify them as patients based on shared numerical patterns. While this may cause temporary anxiety for the patient and lead to costly additional testing, it is medically less dangerous than a false negative because subsequent tests will reveal the true diagnosis, preventing the patient from receiving incorrect treatment without solid confirmation. In our study, we found four cases diagnosed as false positives, demonstrating the model’s ability to learn without being affected by any overfitting.

## Conclusion and future works

This study developed an ML model that achieved accurate and efficient diagnosis using the XGBoost algorithm. It is characterized by the ability to fill in missing data, which helped MICE technology obtain more accurate and logical values for the missing data, and a technique for interpreting model decisions through SHAP to support the interpretation of decisions made by the model for non-technical people, which helps to increase confidence in the model and its decisions. It also implemented Manual Federated Simulation to demonstrate that XGBoost can deliver high performance even when obtaining results from different locations with varying ratios of infected and healthy cases, and to improve the federated model, some standard federated optimization algorithms can be applied to it to obtain a model that is integrated in terms of data confidentiality and security. Furthermore, it implemented data confidentiality measures, as the Global Federated model only processed the results and didn’t view the data.

Integrating different types of data can increase the model’s efficiency and the accuracy of its decisions and also asynchronous Federated Learning where the latest developments are not given to the global model until all local models have completed testing, so for development that by making the model doesn’t wait for all devices from different locations from which new weights are taken, but rather updates are added to the global model as soon as they are available from each location that has finished testing its data, and instead of relying on only two locations to collect data updates, the number of hospitals can be increased which can help in develop the model to determine the best treatment plans for each patient based on their medical reports and test results.

## Data Availability

The datasets analysed during the current study are available in the Mendeley data repository, https://data.mendeley.com/datasets/8wk5hjx7 × 2/1Code Availability Declaration The code is available in zenodo with DOI: 10.5281/zenodo.20805331, https://zenodo.org/records/20805331.

## Code availability

Declaration.

The code is available in zenodo with DOI: 10.5281/zenodo.20805331, https://zenodo.org/records/20805331.
